# The Coexistence of Bicellular and Tricellular Pollen Might Be the Third Type of Pollen Cell Number: Evidence from Annonaceae

**DOI:** 10.3390/biology14050562

**Published:** 2025-05-17

**Authors:** Yangying Gan, Qi Zhang, Chunfen Xiao, Jingyao Ping

**Affiliations:** 1 Institute of Agricultural Economics and Information, Guangdong Academy of Agricultural Sciences/Key Laboratory of Urban Agriculture in South China, Ministry of Agriculture and Rural Affairs, Guangzhou 510640, China; sharelot@163.com; 2 The Agro-Biological Gene Research Center, Guangdong Academy of Agricultural Sciences, Guangzhou 510640, China; zhangqi@agrogene.ac.cn; 3 Xishuangbanna Tropical Botanical Garden, Chinese Academy of Sciences, Xishuangbanna 666303, China; xiaocf@xtbg.ac.cn; 4 College of Life Sciences, Sun Yet-Sen University, Guangzhou 510275, China

**Keywords:** Annonaceae, pollen cell number, bicellular pollen, tricellular pollen

## Abstract

Anther is thought to release either bicellular or tricellular pollen when mature. Though a few species had been found to shed both bicellular and tricellular pollen, due to their low frequency, they had been overlooked as special cases of bicellular or tricellular pollen in previous phylogenetic studies. In the present work, the pollen cytologies of 89 species from 26 genera of Annonaceae were observed using the overall transparency method and paraffin sectioning method. The results show that 73 species from 25 genera distribute bicellular pollen, while 16 species from 10 genera shed both bicellular and tricellular pollen. This is the first time that so many species with both types of pollen has been observed in the same family. Combined with reports from other families, the plants that were known to shed both types of pollen included 15 families, 40 genera, and 52 species. Our results indicate that the coexistence of bicellular and tricellular pollen might be the third type of pollen cell number. And the systematic relationship among them is needed to be reanalyzed.

## Simple Summary

Anther is thought to release either bicellular or tricellular pollen when mature. In the present work, we found that 16 species from 10 genera of Annonaceae shed both bicellular and tricellular pollen. This is the first time that so many species with both types of pollen has been observed in the same family. Combined with reports from other families, the plants that were known to shed both types of pollen included 15 families, 40 genera, and 52 species. Our results indicate that the coexistence of bicellular and tricellular pollen might be the third type of pollen cell number. And the systematic relationship among them is needed to be reanalyzed.

## 1. Introduction

Most angiosperms contain only one vegetative cell and one reproductive cell in their pollen before dispersal, known as bicellular pollen. About 30% of angiosperms complete the second cell division before dispersal, forming two reproductive cells and one vegetative cell, known as tricellular pollen [1]. There has long been controversy regarding the systematic evolution of pollen cell numbers. Early researchers believed that bicellular pollen was primitive and that the evolution from bicellular to tricellular pollen was irreversible [2,3]. Based on these claims, Webster and Rupert proposed the “Schürhoff–Brewbaker Law” [4]. However, subsequent research has suggested that the evolution from bicellular to tricellular pollen is reversible, and is skeptical of the primitive trait [1]. Additionally, in all previous systematic analyses on pollen cell numbers, only bicellular and tricellular pollen were taken into consideration. Although a few species had been found to shed both types of pollen [5,6,7], due to their low frequency, they had been considered as special cases and excluded from samples [1,3]. But, over time, more and more species with both kinds of pollen have been discovered [8,9,10,11,12,13,14,15,16].

Annonaceae was reported to be bicellulate by Brewbaker [3], but the coexistence of bicellular and tricellular pollen has been found in anthers of *Annona cherimola* Mill. and *Mitrephora macclurei* [6,9]. According to previous reports, Annonaceae has great diversity in terms of pollen size, shape, polarity, symmetry, dispersal unit, number/position/shape of germination aperture, ornamentation, and tectal and infratectal characters [17,18,19,20]. As a large family, comprising 107 genera and c.2400 species [21], it may also be diverse in terms of pollen cell number. But, until now, only nine species from six genera are known to have bicellular pollen [1,3,22,23,24,25], and two species from two genera are known to have both types of pollen [6,9,26]. Among the other undetected species, will there be more abundant discoveries (more taxa with both types of pollen, or even tricellular pollen)? With these questions and expectations, we have observed the pollen cell numbers of most of the Annonaceae plants currently distributed or introduced in China. The results may enrich our botanical understanding of pollen cell numbers and provide more evidence for systematic evolution research.

## 2. Materials and Methods

### 2.1. Materials

Fully mature flowers from 89 species across 26 genera of Annonaceae were collected from 2019 to 2021, taking about 5–10 flowers per plant and 5–10 anthers per flower annually for 2–3 consecutive years. The sampling species information includes the location, introduction number, and specimen number. The introduction number is available on the official website of the three botanical gardens (Xishuangbanna Tropical Botanical Garden: https://www.xtbg.ac.cn/ (accessed on 15 March 2024); Wuhan Botanical Garden: http://www.whiob.ac.cn/ (accessed on 18 March 2024); South China Botanical Garden: https://www.scbg.ac.cn/ (accessed on 20 March 2024)). The specimen number is available on the official website of the National Plant Specimen Resource Center of China (http://www.nsii.org.cn (accessed on 22 March 2024)) or the Chinese Virtual Herbarium (https://www.cvh.ac.cn/ (accessed on 25 March 2024)). All materials were fixed in formalin acetic alcohol (FAA: 70% alcohol, formaldehyde, and glacial acetic acid in a ratio of 90:5:5).

### 2.2. Methods

The pollen cell numbers were observed either by the overall transparency method described by Fu et al. [27] or the paraffin sectioning method described by Gan and Xu [9]. The proportion of anthers with both types of pollen in the sample anthers was also calculated.

Overall transparency method: All pollen was peeled off from mature anthers under a dissecting microscope to create a pollen suspension. The suspension was then subjected to hydrochloric acid hydrolysis, hematoxylin staining, gradient alcohol dehydration (30%, 50%, 70%, 90%, 95%, 100%) and transparent treatment with methyl salicylate, followed by DAPI staining, and placed on a glass slide dripped with clove oil for sealing. Fluorescence microscopy was used for observation and photography.

Paraffin sectioning method: For 64 species that the overall transparency method was not applicable, the paraffin sectioning method described by Gan and Xu [9] was applied instead. FAA-fixed anthers were subjected to ethanol gradient dehydration, xylene transparency, safranin-fixed green staining, paraffin embedding, and sectioning for 9 μm. A Leica DFC550 optical microscope (Leica Microsystem, Wetzlar, Germany) was used for observation and photography.

## 3. Results

**Figure 1:**
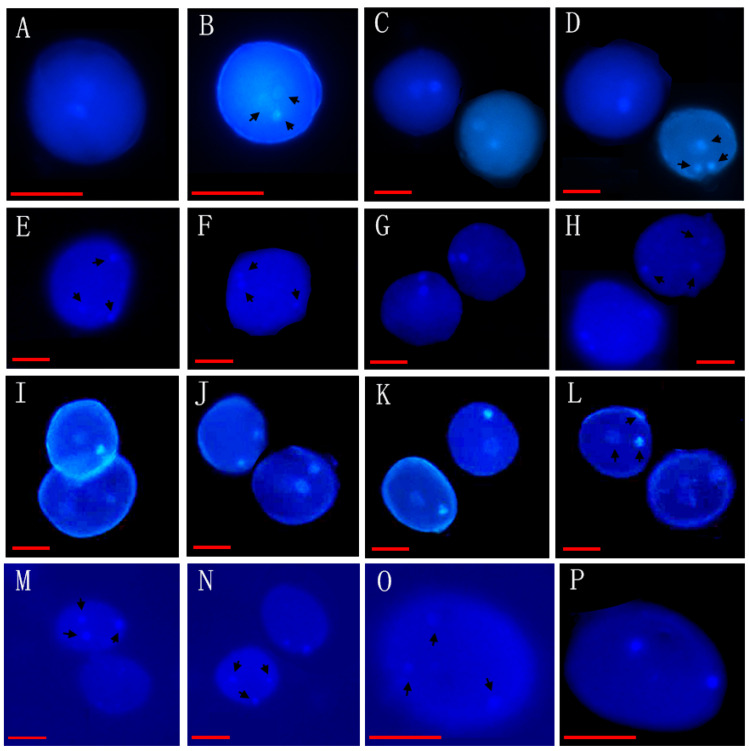
Mixed developmental stages of Annonaceae pollen before anther release (I). (**A**–**D**) The bicellular (**A**,**C**,**D left**) and tricellular (**B**,**D right**) pollen of *Uvaria grandiflora* Roxb; (**E**–**H**) the bicellular (**G**) and tricellular (**E**,**F**,**H**) pollen of *Uvaria kurzii* (King) P. T. Li; (**I**–**L**) the bicellular (**I**–**K**) and tricellular (**L**) pollen of *Artabotrys hexapetalus* (L. f.) Bhandar; (**M**–**P**) the bicellular (**N right**, **P**) and tricellular (**M**,**N left**,**O**) pollen of *Artabotrys hongkongensis* Hance. Scale bar = 50 μm. Arrows show cell nucleus.

**Figure 2:**
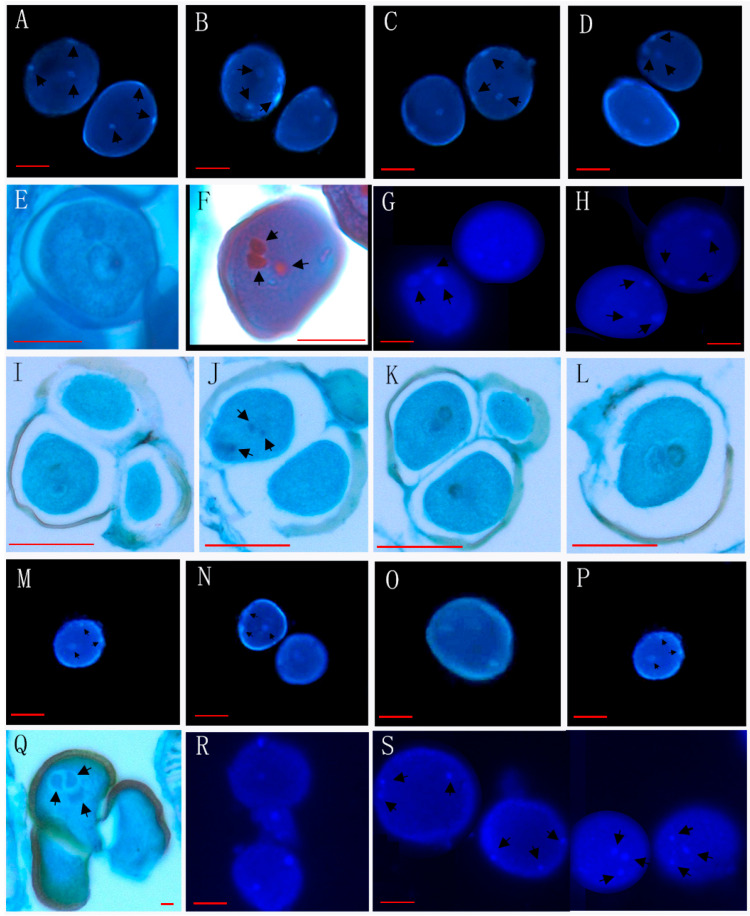
Mixed developmental stages of Annonaceae pollen before anther release (II). (**A**–**D**) The bicellular (**C left**,**D right**) and tricellular (**A**,**B**,**C right**,**D right**) pollen of *Artabotrys pachypetalus* B. Xue & Junhao Chen; (**E**–**H**) the bicellular (**E**) and tricellular (**F**–**H**) pollen of *Goniothalamus calvicarpus* Craib; (**I**–**L**) the bicellular (**I**,**J down**,**K**,**L**) and tricellular (**J up**) pollen of *Goniothalamus gardneri* Hook. f. et Thoms; (**M**–**P**) the bicellular (**N right**, **O**) and tricellular (**M**,**N left**,**P**) pollen of *Fissisfigma polyanthum* Hook. f. et Thoms; (**Q**) the tricellular pollen of *Annona cherimola* Mill; (**R**,**S**) the bicellular (**R**) and tricellular (**S**) pollen of *Uvaria calamistrata* Hance. Scale bar = 50 μm. Arrows show cell nucleus.

**Figure 3:**
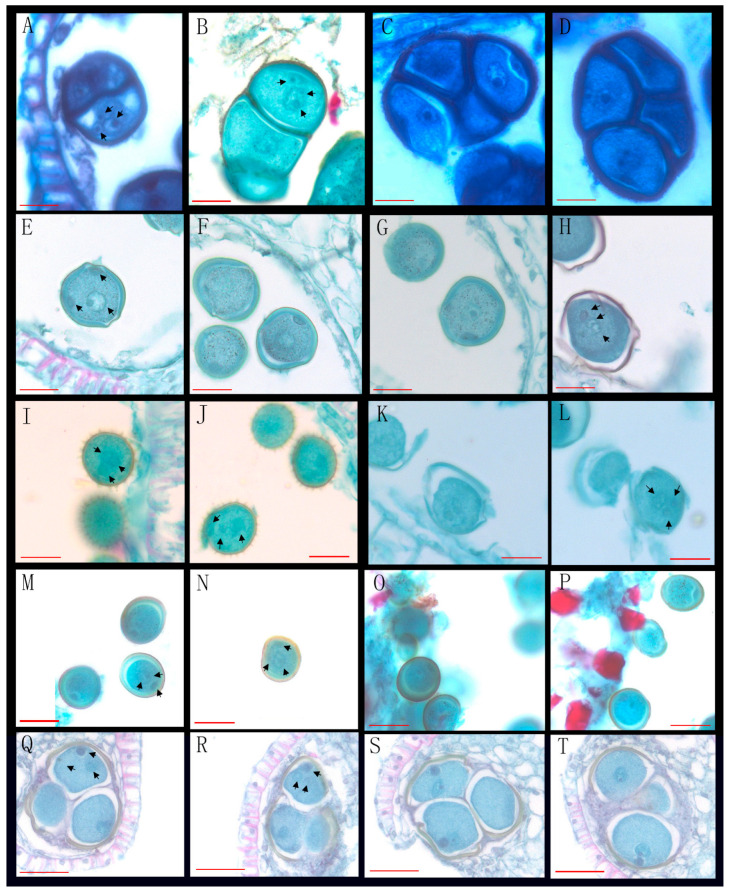
Mixed developmental stages of Annonaceae pollen before anther release (III). (**A**–**D**) The bicellular (**A up**,**B down**,**C**,**D**) and tricellular (**A down**,**B up**) pollen of *Mitrephora wangii* Hu; (**E**–**H**) the bicellular (**F**,**G**) and tricellular (**E**,**H**) pollen of *Meiogyne oligocarpa* B. Xue & Y. H. Ta; (**I**,**J**) the bicellular (**J up**) and tricellular (**I**,**J down**) pollen of *Dasymaschalon rostratum* Merr. & Chun; (**K**,**L**) the bicellular (**K**) and tricellular (**L**) pollen of *Orophea laui* Leonardía & Kessler; (**M**–**P**) the tricellular (**M right**,**N**) and bicellular (**O**,**P**) pollen of *Polyalthia cheliensis* Hu; (**Q**–**T**) the tricellular (**Q**,**R**) and bicellular (**S**,**T**) pollen of *Goniothalamus chinensis* Merr. et Chun. Scale bar = 50 μm. Arrows show cell nucleus.

**Figure 4:**
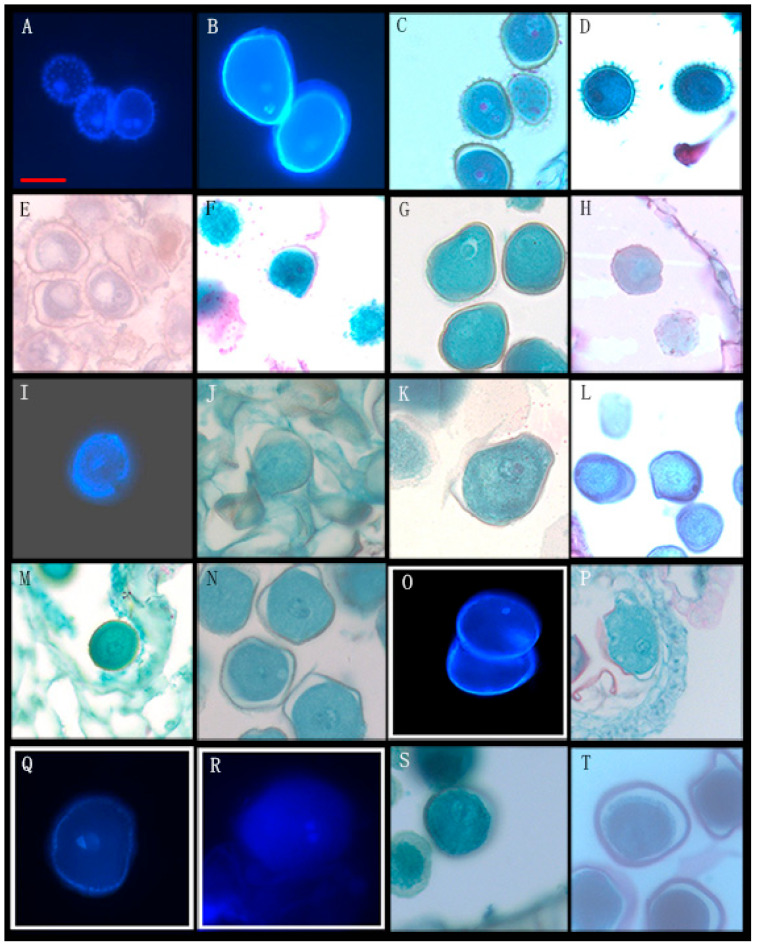
Bicellular pollen of Annonaceae shortly before anther dehiscence. (I). (**A**) *Desmos chinensis* Lour; (**B**) *Desmos dumosus* (roxb.) saff; (**C**) *Desmos yunnanensis* (Hu) P. T. Li; (**D**) *Dasymaschalon trichophorum* Merr; (**E**) *Desymaschalon filipes* (Ridl.) Ridl.Ban; (**F**) *Dasymaschalon macrocalyx* Finet & Gagnep; (**G**) *Polyalthia suberosa* (Roxburgh) Thwaites; (**H**) *Polyalthia verrucipes* C. Y. Wu ex P. T. Li; (**I**) *Polyalthia laui* Merrill; (**J**) *Polyalthia chinensis* S. K. Wu & P. T. L; (**K**) *Polyalthia yingjiangensis* Y. H. Tan & B. Xue; (**L**) *Polyalthia obliqua* Hook.f. & Thomson; (**M**) *Polyalthia longifolia* (Sonn.) Thwaites; (**N**) *Hubera cerasoides* (Roxb.) Benth.et Hook.f.ex Bedd; (**O**) *Cananga odorata* (Lamarck) J. D. Hooker & Thomson; (**P**) *Cananga odorata* var. fruticosa (Craib) J.Sinclair; (**Q**) *Alphonsea monogyna* Merrill & Chun, (**R**) *Alphonsea mollis* Dunn; (**S**) *Alphonsea glandulosa* Y.H. Tan & B. Xue; (**T**) *Alphonsea ventricosa* (Roxb.) Hook.f.&Thomson. Scale bar = 50 μm.

**Figure 5:**
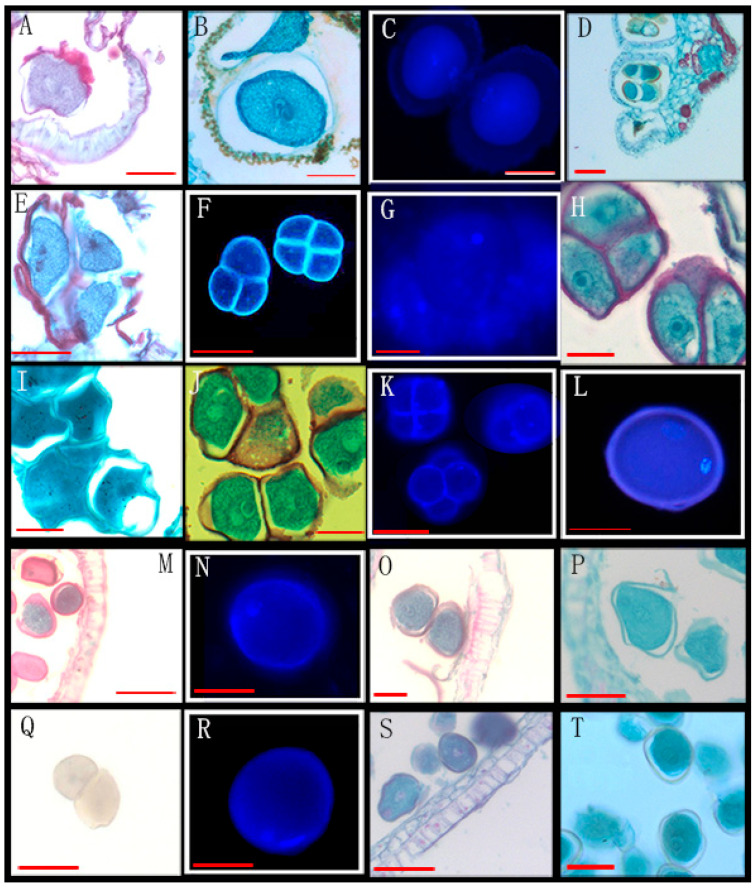
Bicellular pollen of Annonaceae shortly before anther dehiscence. (II). (**A**) *Annona squamosa* Linn; (**B**) *Annona muricata* Linnaeus; (**C**) *Annona montana* Macf; (**D**) *Annona reticulata* L; (**E**) *Annona glabra* Linn; (**F**) *Mitrephora thorelii* Pierre; (**G**,**H**) *Mitrephora teysmannii* Scheff; (**I**) *Mitrephora sirikitiae* Weeras; (**J**,**K**) *Pseuduvaria trimera* (Craib) Y. C. F. Su & R. M. K. Saunders; (**L**) *Artabotrys hainanensis* R. E. Fries; (**M**) *Artabotrys pilosis* Merrill & Chun; (**N**,**O**) *Trivalvaria costata* (J. D. Hooker & Thomson) I. M. Turner; (**P**) *Trivalvaria carnosa* (Teijsm. & Binn.) Scheff; (**Q**) *Uvaria yunnanensis* Hu; (**R**) *Marsypopetalum littorale* (Bl.) B. Xue & R. M. K; (**S**) *Chieniodendron hainanense* (Merr.) Tsiang et P. T. Li; (**T**) *Cleistopholis glauca* Pierre ex Engl. & Diels. Scale bar = 50 μm.

**Figure 6:**
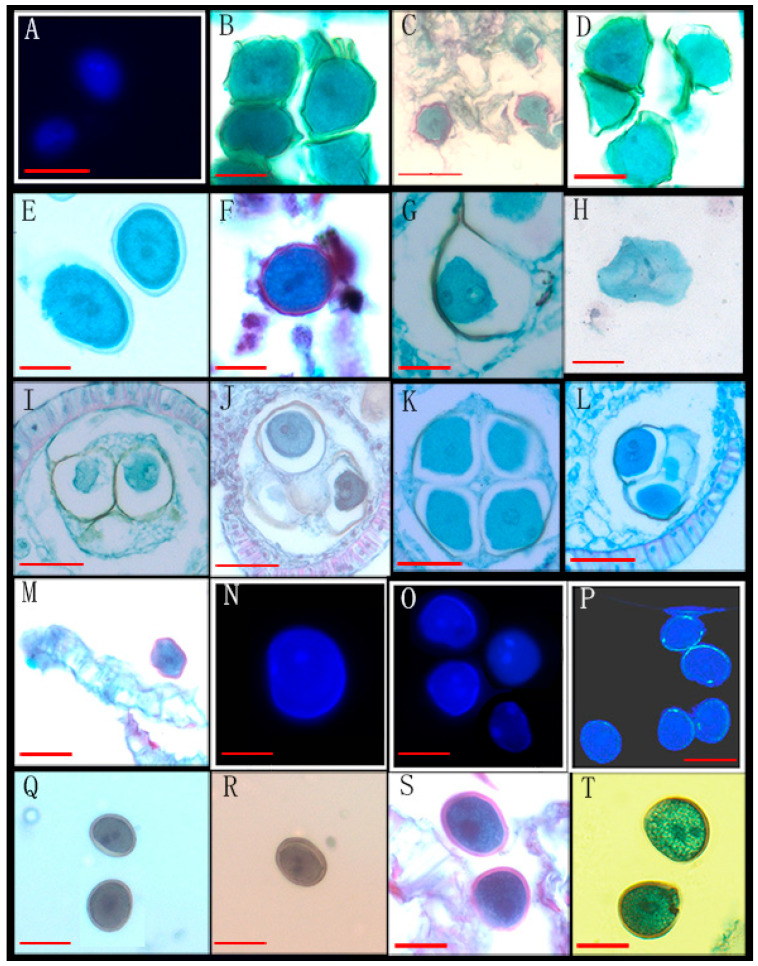
Bicellular pollen of Annonaceae shortly before anther dehiscence. (III). (**A**) *Uvaria macrophylla* Roxb; (**B**) *Uvaria tokinensis* Finet et Gagnep; (**C**) *Uvaria tonkinensis* var. subglabra Melodorum; (**D**) *Uvaria kweichowensis* P. T. Li; (**E**) *Uvaria grandiflora* var.flava (Teijsm. & Binn.) Scheff; (**F**) *Uvaria rufa* Bl; (**G**) *Goniothalamus saccopetaloides* Y.H. Tan & Bin Yang; (**H**) *Uvaria boniana* Finet et Gagnep; (**I**) *Goniothalamus howii* Merrill & Chun; (**J**) *Goniothalamus donnaiensis* Finet et Gagnep; (**K**) *Goniothalamus cheliensis* Hu; (**L**) *Goniothalamus leiocarpus* (W. T. Wang) P. T. Li; (**M**) *Fissistigma acuminatissimum* Merrill; (**N**) *Fissistigma polyanthoides* (Aug. DC.) Merr; (**O**) *Fissistigma glaucescens* (Hance) Merrill; (**P**) *Fissistigma wallichii* (Hook. f. et Thoms.) Merr; (**Q**) *Fissistigma bracteolatum* Chatt; (**R**) *Fissistigma maclurei* Merr; (**S**) *Fissistigma uonicum* (Dunn) Merr; (**T**) *Fissistigma thorelii* (Pierre ex Finet&Gagnep.) Merr. Scale bar = 50 μm.

**Figure 7:**
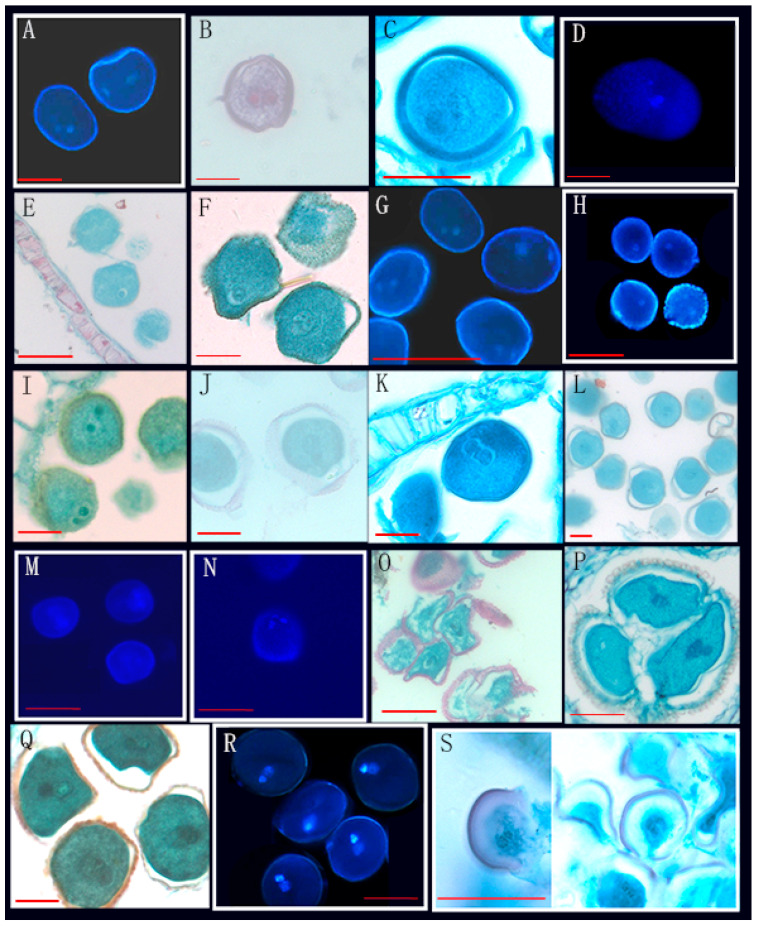
Bicellular pollen of Annonaceae shortly before anther dehiscence. (IV). (**A**,**B**) *Orophea hainanensis* Merr; (**C**) *Orophea hirsuta* King; (**D**) *Anaxagorea luzonensis* A. Gray; (**E**) *Anaxagorea javanica* Blume; (**F**) *Miliusa chunii* W. T. Wan; (**G**) *Miliusa horsfieldii* (Bennett) Pierre; (**H**,**I**) *Miliusa sinensis* Finet et Gagnep; (**J**) *Miliusa chantaburiana* Damthongdee & Chaowasku; (**K**) *Miliusa glochidioides* Hand.-Mazz; (**L**) *Miliusa bannaensis* X.L. Hou; (**M**) *Melodorum fruticosum* Lour; (**N**) *Melodorum siamense* (Scheff.) Bân; (**O**,**Q**) *Asimina triloba* Dunal; (**P**) *Disepalum plagioneurum* (Diels) D. M. Johnson; (**R**) *Popowia pisocarpa* (Bl.) Endl. in Walp. Rep; (**S**) *Rollinia mucosa* (Jacquin) Baillon. Scale bar = 50 μm.

Through the observation of 89 species from 26 genera of Annonaceae, we found that 16 species from 10 genera disperse both types of pollen (Figure 1, Figure 2 and Figure 3, Table 1), while 73 species from 25 genera shed bicellular pollen (Figure 4, Figure 5, Figure 6 and Figure 7, Table 1). Among the 16 species that disperse both types of pollen, 12 of them had more than half of anthers that contain both types of pollen, and for others such as *Uvaria grandiflora* Roxb, *Uvaria calamistrata* Hance, *Mitrephora wangii* Hu and *Artabotrys hexapetalus* (L. f.) Bhandar, 20–30% of sample anthers generally had both types of pollen. Our findings increase the number of known plants in Annonaceae that disperse both types of pollen from 2 genera and 2 species to 10 genera and 17 species (Table 2), However, pure tricellular pollen has not yet been detected in this family. In this study, we also observed both types of pollen (Figure 2Q) in the anthers of *Annona cherimola* Mill., confirming the research report of Lora et al. [26]. Additionally, we once again observed both types of pollen within the same pollen unit (Figure 2I–L), supporting the report by Gan and Xu [9].

**Figure 8:**
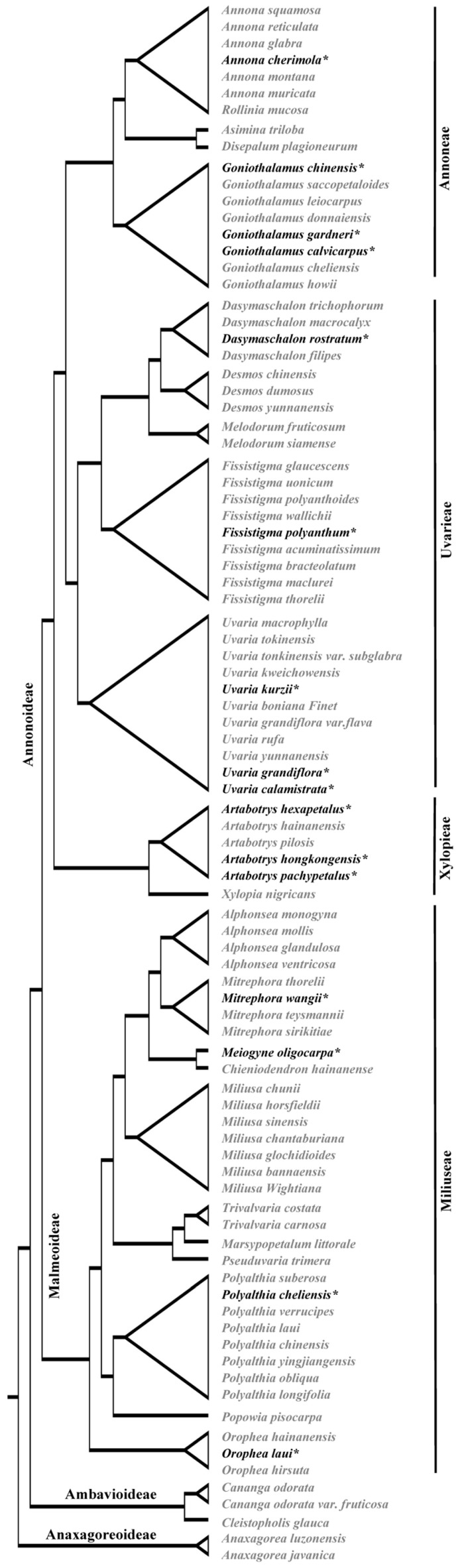
The phylogenetic relationships of sampled species at the genus level. Species with both types of pollen are black and marked with *; others are species with binucleate pollen. The phylogenetic tree referenced Guo et al. [21].

According to the molecular phylogenetic tree of the Annonaceae family constructed by Guo et al. [21], we found that species with both types of pollen are mostly distributed in the relatively evolved tribes, including *Annoneae*, *Uvariaeae* (Annonoideae), and *Miliuseae* (Malmeoideae) (Figure 8). No samples containing both types of pollen were found in the more primitive subfamilies, such as Anaxagoreoideae and Ambavioideae.

## 4. Discussion

### 4.1. The Coexistence of Bicellular and Tricellular Pollen May Be More Prevalent in Angiosperms than We Thought

Grayum [5] and Lora et al. [6] reported that the coexistence of bicellular and tricellular pollen occurs transiently prior to dispersal, often with an imbalanced ratio of the two pollen types. If samples are collected prematurely, this mixed population may be misinterpreted as exclusively bicellular pollen. Similarly, incomplete sampling could lead to misclassification as either purely bicellular or tricellular pollen. Such errors have historically caused taxonomic inconsistencies. For example, *Calla palustris* was described as tricellular by Dudley [41] but bicellular by Brewbaker [3]; subsequent detailed analysis by Grayum [5] revealed that ~5% of pollen completed the second mitotic division prior to dispersal, confirming mixed development. Similarly, while Annonaceae was initially classified as bicellular [3], Rosell et al. [42] identified tricellular pollen in *Annona cherimola*. later corroborated by Lora et al. [6], who observed both pollen types 9–10 h before anther dehiscence. To mitigate sampling bias, our study exclusively used fully matured flowers at the point of pollen dispersal. We confirmed mixed bicellular and tricellular pollen in *Mitrephora maingayi* Hook. f. et Thoms [9] and 16 additional species across 10 genera within Annonaceae. This suggests that strict developmental staging of floral material is critical, and we hypothesize that broader sampling with rigorous temporal controls may reveal this phenomenon to be more widespread in angiosperms than currently acknowledged.

### 4.2. Is the Coexistence of Bicellular and Tricellular Pollen a Special Case or the Third Type?

Previous systematic analysis on pollen cell numbers has treated mixed populations as anomalies of either bicellular or tricellular pollen, often excluding them from samples [1,4,43]. However, within Annonaceae, 17 of 90 species (~19%) and 10 of 30 genera (~33%) exhibit this trait [1,3,22,23,24,25]. Notably, the ratio of bicellular and tricellular pollen is not always imbalanced. A high percentage of both pollen types have been observed in six species of Araceae [5]. Lora et al. [6] reported a mixed population of bicellular (49%) and tricellular (51%) pollen in *Annona cherimola* Mill. In our study, some anthers contained up to 40% tricellular pollen. In these cases, distinguishing whether this represents a variant of bicellular or tricellular pollen becomes challenging. Thus, at least within Annonaceae, it may be reasonable to take the coexistence of bicellular and tricellular as the third type except for bicellular and tricellular pollen.

In recent years, the discovery of anthers containing both types of pollen has continued to increase in other families, such as the *Arundinaria simonii f.albostriatus* and *Shibataea chinensis* Nakai [36], the *Bambusa textilis* [14], *Sasaella kogasensis* ‘Aureostriatus’ [11], *Bambusa multiplex* [8], *Pseudosasa viridula* [12], *Menstruocalamus sichuanensis* [36] and *Phyllostachys edulis* (Carrière) J. Houzeau [16] from Poaceae, the *Swertia bimaculata* [31] and *Tripterospermum chinense* (Migo) Harry Sm. [32] from Gentianaceae, the *Limonium* from Plumbaginaceae [35], which were previously reported as tricellular [3,44]. Similarly, *Conyza canadensis* (L.) C ronq [29] from Asteraceae, *Michelia figo* (Lour.) Spreng from Magnoliaceae [15], *Coptis deltoidea* C. Y. Cheng et Hsiao [37] and *Adonis amurensis* Regel et Radde [10] from Ranunculaceae, *Viola tricolor* L. from Violaceae [10], *Diphylleia sinensis* H. L. Li [30] and *Leontice incerta* Pall. [12] from Berberidaceae, *Solanum japonense* Nakai and *Solanum septemlobum* Bunge from Solanaceae [39], were previously reported as bicellular [1,3]. Additionally, there is also *Hemerocallis* from Asphodelaceae [28], which had not been reported before. Table 2 lists all the reports of plants shedding both types of pollen. These findings support the idea of treating the coexistence of both types of pollen as the third type of pollen cell number.

### 4.3. Which Is Primitive and How It Evolved?

The systematic study of pollen cell numbers has been developed and controversial for over a century. Schürhoff [45] firstly observed that most plant families produce either bicellular or tricellular pollen. Schnarf [46,47,48] and Brewbaker [3] further noted that taxa with bicellular pollen were predominantly basal within the phylogenetic tree. Webster and Rupert [4] proposed the “Schiirhoff-Brewbaker Law”, which claims that bicellular pollen was primitive in the angiosperms as a whole and that the evolution process from bicellular to tricellular pollen was irreversible. However, this hypothesis was challenged by Gardner [34], who identified tricellular pollen in Lauraceae (a primitive group), and Webster et al. [49], who found tricellular pollen in early-diverging Euphorbieae. Williams et al. [1] analyzed 2511 species and modeled trait evolution using time-calibrated phylogenines, revealing reversible transitions between bicellular and tricellular states, as well as differential diversification rates between the two lineages. Although they questioned the likelihood of a tricellular origin, they could not conclusively determine the ancestral state.

Interestingly, gymnosperms exhibit varying mitotic divisions during pollen maturation, releasing pollen at different male gametophyte developmental stages [50]. However, previous systematic studies on angiosperms pollen have only considered strictly bicellular and tricellular forms, often disregarding mixed cases as anomalies. If the coexistence of both types of pollen are taken into account, where should it be located? From the limited known results (Table 2), the coexistence of both types of pollen are found in primitive families such as Lauraceae, Magnoliaceae, Annonaceae, and relatively advanced families such as Asteraceae, Gentianaceae and Violaceae. However, in Annonaceae, the coexistence of both types of pollen are mostly distributed in relatively advanced tribes such as Annoneae, Uvariaeae (Annonoideae), and Miliuseae (Malmeoideae) (Figure 8). Lora et al. [6] indicated that the coexistence of both types of pollen may have stronger adaptability than bicellular and tricellular pollen because the environmental conditions of pollen after dispersal are unpredictable, and the combined type of pollen could increase the chance of fertilization. Thus, the coexistence of both types of pollen may be advanced.

Franchi et al. [51] proposed that pollen, like seeds, may undergo developmental arrest (DA) under unfavorable conditions. DA in pollen refers to the phenomenon where the development of pollen slows or temporarily halts before reaching full maturity. This process typically occurs at the bicellular stage, during which the pollen consists of one vegetative cell and one generative cell that has not yet divided into two sperm cells, such as drought conditions Furthermore, DA can occur at any stage of pollen development; it might manifest as bicellular pollen when arrested at the bicellular stage, as either type of pollen when halted during the transition from bicellular to tricellular stage, or as tricellular pollen when environment conditions are favorable and no DA takes place. DA in pollen is strongly associated with the acquisition of desiccation tolerance (DT), which extends pollen viability during air travel [52]. Williams and Brown [53] found a close relationship between the number of pollen cells and pollen water content, revealing that most tricellular pollen exhibited a relatively high water content, whereas bicellular pollen showed comparatively low water content. From the perspective of DA, bicellular pollen appears to represent an evolutionary adaptation from aquatic to terrestrial plants. Additionally, the retrogradation of tricellular pollen observed by Williams et al. [1] may have resulted from a loss of dehydration tolerance during subsequent evolutionary processes.

## 5. Conclusions

In the present study, we identified 16 species from 10 genera of Annonaceae that shed both types of pollen. Including reports from other families, approximately 15 families, 40 genera, and 52 species are known to produce both types of pollen. The coexistence of bicellular and tricellular pollen might be the third type of pollen cell number. And the systematic relationship among them is needed to be reanalyzed.

## Figures and Tables

**Table 1:** List of investigated species with provenance, voucher number and corresponding figure plate.

No.	Taxon	Provenance ^a^	Voucher	Figures
1	*Desmos chinensis* Lour.	SCBG	xx060308, 20081072, xx271155	Figure 4A
2	*Desmos dumosus* (roxb.)saff.	XTBG	00012542, C06009, 273677	Figure 4B
3	*Desmos yunnanensis* (Hu) P. T. Li	XTBG	275056, 258389	Figure 4C
4	*Dasymaschalon trichophorum* Merr.	SCBG	20011172, 19975026, 19970018	Figure 4D
5	*Dasymaschalon filipes* (Ridl.) Ridl.Ban	XTBG	1320030093, 0020220650	Figure 4E
6	*Dasymaschalon rostratum* Merr. & Chun *	XTBG	0020023226, 0020020479	Figure 3I,J
7	*Dasymaschalon macrocalyx* Finet & Gagnep.	XTBG	1320030078, 0020022105	Figure 4F
8	*Polyalthia suberosa* (Roxburgh) Thwaites	SCBG	20010982, 20070804, 20140665	Figure 4G
9	*Polyalthia cheliensis* Hu *	XTBG	0020081058, 0020100741	Figure 3M–P
10	*Polyalthia verrucipes* C. Y. Wu ex P. T. Li	XTBG	0020031897(2)	Figure 4H
11	*Polyalthia laui* Merrill	SCBG	xx240010, xx320026	Figure 4I
12	*Polyalthia chinensis* S. K. Wu & P. T. Li	XTBG	0020023088, 0020150274	Figure 4J
13	*Polyalthia yingjiangensis* Y. H. Tan and B. Xue	XTBG	0020021384(3)	Figure 4K
14	*Polyalthia obliqua* Hook.f. & Thomson	XTBG	0020013801(3)	Figure 4L
15	*Polyalthia longifolia* (Sonn.) Thwaites	SCBG	xx271140, 20055086	Figure 4M
16	*Hubera cerasoides* (Roxb.) Benth.et Hook.f.ex Bedd.	SCBG	20031137(3)	Figure 4N
17	*Annona squamosa* Linn.	XTBG	0020071074(3)	Figure 5A
18	*Annona muricata* Linnaeus	SCBG	19940242, 20070500	Figure 5B
19	*Annona montana* Macf	XTBG	0019600558A, 2019940014	Figure 5C
20	*Annona reticulata* L.	XTBG	0320140001, 275081	Figure 5D
21	*Annona glabra* Linn.	SCBG	xx080063, xx080276, xx080383	Figure 5E
22	*Annona cherimola* Mill. *	XTBG	1520060007(3)	Figure 2Q
23	*Cananga odorata* (Lamarck) J. D. Hooker & Thomson	SCBG	20090663, 20140876, xx120033	Figure 4O
24	*Cananga odorata var. fruticosa* (Craib) J.Sinclair	SCBG	20040790(3)	Figure 4P
25	*Mitrephora thorelii* Pierre	SCBG	20030719(3)	Figure 5F
26	*Mitrephora wangii* Hu *	XTBG	0020022041, 256509, 0019780252	Figure 3A–D
27	*Mitrephora teysmannii* Scheff	SCBG	20042613(3)	Figure 5G,H
28	*Mitrephora sirikitiae* Weeras	XTBG	3820130137(3)	Figure 5I
29	*Pseuduvaria trimera* (Craib) YCF Su & RMK. Saunders	XTBG	0019880077, 274490, 3020020011	Figure 5J,K
30	*Alphonsea monogyna* Merrill & Chun	SCBG	20011014(3)	Figure 4Q
31	*Alphonsea mollis* Dunn	XTBG	0020040356, 0020150149, 286072	Figure 4R
32	*Alphonsea glandulosa* Y.H. Tan & B. Xue	XTBG	0019750173(3)	Figure 4S
33	*Alphonsea ventricosa* (Roxb.) Hook.f.&Thomson	XTBG	0019970165(3)	Figure 4T
34	*Artabotrys hexapetalus* (L. f.) Bhanda *	XTBG	0020040193, 0020090146	Figure 1I–L
35	*Artabotrys hainanensis* R. E. Fries	SCBG	20012148(3)	Figure 5L
36	*Artabotrys pilosis* Merrill & Chun	SCBG	00012542(3)	Figure 5M
37	*Artabotrys hongkongensis* Hance *	SCBG	20011052(3)	Figure 1M–P
38	*Artabotrys pachypetalus* B.Xue & Junhao Chen *	SCBG	00028772(3)	Figure 2A–D
39	*Trivalvaria costata* (J. D. Hooker & Thomson) I. M. Turner	SCBG	xx110217(3)	Figure 5N,O
40	*Trivalvaria carnosa* (Teijsm. & Binn.) Scheff	XTBG	1320010126(3)	Figure 5P
41	*Uvaria macrophylla* Roxb	XTBG	0020023255, 287484, 0020090148	Figure 6A
42	*Uvaria grandiflora* Roxb *	XTBG	3820021106(3)	Figure 1A–D
43	*Uvaria calamistrata* Hance *	XTBG	0020201075(3)	Figure 2R,S
44	*Uvaria tokinensis* Finet et Gagnep	XTBG	3820020732(3)	Figure 6B
45	*Uvaria tonkinensis var. subglabra* Melodorum	SCBG	20030552(3)	Figure 6C
46	*Uvaria kweichowensis* P. T. Li	SCBG	20031112(3)	Figure 6D
47	*Uvaria kurzii* (King) P. T. Li *	SCBG	042778(3)	Figure 1E–H
48	*Uvaria boniana* Finet et Gagnep	SCBG	00044133(3)	Figure 6H
49	*Uvaria grandiflora var. flava* (Teijsm. & Binn.) Scheff	XTBG	3820130135(3)	Figure 6E
50	*Uvaria rufa* Bl	XTBG	284407(3)	Figure 6F
51	*Uvaria yunnanensis* Hu	XTBG	0020070685(3)	Figure 5Q
52	*Marsypopetalum littorale* (Bl.) B. Xue & R. M. K.	XTBG	0020012213(3)	Figure 5R
53	*Goniothalamus chinensis* Merr. et Chun *	XTBG	3020021381, 0020162454	Figure 3Q–T
54	*Goniothalamus calvicarpus* Craib *	SCBG	20042665, 284928, 275874	Figure 2E–H
55	*Goniothalamus gardneri* Hook. f. et Thoms *	SCBG	20113045(3)	Figure 2I–L
56	*Goniothalamus saccopetaloides* Y.H. Tan and Bin Yang	XTBG	3020020407(2)	Figure 6G
57	*Goniothalamus cheliensis* Hu	XTBG	3020050005, C30121, 274125	Figure 6K
58	*Goniothalamus leiocarpus* (W. T. Wang) P. T. Li	XTBG	0020013790(2)	Figure 6L
59	*Goniothalamus howii* Merrill & Chun	XTBG	0020021240(2)	Figure 6I
60	*Goniothalamus donnaiensis* Finet et Gagnep	LMNR	AU072086 ^b^	Figure 6J
61	*Fissistigma wallichii* (Hook. f. et Thoms.) Merr	XTBG	0020070161, 0020100719	Figure 6P
62	*Fissistigma glaucescens* (Hance) Merrill	SCBG	XX271312(2)	Figure 6O
63	*Fissistigma polyanthum* Hook. f. et Thoms *	SCBG	20050538(2)	Figure 2M–P
64	*Fissistigma polyanthoides* (Aug. DC.) Merr.	XTBG	0020011877, 275869, 275870	Figure 6N
65	*Fissistigma acuminatissimum* Merrill	XTBG	285650(2)	Figure 6M
66	*Fissistigma bracteolatum* Chatt	XTBG	374189, 274189, 277352	Figure 6Q
67	*Fissistigma maclurei* Merr	XTBG	0020080638, 286008, 286009	Figure 6R
68	*Fissistigma uonicum* (Dunn) Merr	NMNR	0078738 ^b^	Figure 6S
69	*Fissistigma thorelii* (Pierre ex Finet&Gagnep.) Merr	XTBG	0020020480, 0020031109	Figure 6T
70	*Meiogyne oligocarpa* B. Xue & Y. H. Tan *	XTBG	0020013864(2)	Figure 3E–H
71	*Chieniodendron hainanense* (Merr.) Tsiang et P. T. Li	SCBG	19980193(2)	Figure 5S
72	*Cleistopholis glauca* Pierre ex Engl. & Diels	XTBG	3119800151(2)	Figure 5T
73	*Orophea hainanensis* Merr	SCBG	20011196(3)	Figure 7A,B
74	*Orophea laui* Leonardía & Kessler *	XTBG	275549, 287159	Figure 3K,L
75	*Orophea hirsuta* King	SCBG	20011196(2)	Figure 7C
76	*Anaxagorea luzonensis* A. Gray	DMNR	1020170057(2)	Figure 7D
77	*Anaxagorea javanica* Blume	XTBG	3820021060, 0020200920	Figure 7E
78	*Miliusa chunii* W. T. Wan	XTBG	0019970179(2)	Figure 7F
79	*Miliusa horsfieldii* (Bennett) Pierre	SCBG	20051897(2)	Figure 7G
80	*Miliusa sinensis* Finet et Gagnep	SCBG	011047(2)	Figure 7H,I
81	*Miliusa chantaburiana* Damthongdee & Chaowasku	XTBG	0020210589(2)	Figure 7J
82	*Miliusa glochidioides* Hand.-Mazz.	SCBG	20113642(2)	Figure 7K
83	*Miliusa bannaensis* X.L. Hou	XTBG	0020060634(2)	Figure 7L
84	*Melodorum fruticosum* Lour	XTBG	3820021019(2)	Figure 7M
85	*Melodorum siamense* (Scheff.) Bân	XTBG	3820021101(2)	Figure 7N
86	*Disepalum plagioneurum* (Diels) D. M. Johnson	DMNR	01187407 ^b^	Figure 7P
87	*Popowia pisocarpa* (Bl.) Endl. in Walp. Rep	DMNR	0079742 ^b^	Figure 7R
88	*Asimina triloba* Dunal.	WBG	20177223(2)	Figure 7O,Q
89	*Rollinia mucosa* (Jacquin) Baillon	SCBG	AU080617 ^b^	Figure 7S

Taxoxon with “*” have both types of pollen; others are binucleate. ^a^ SCBG (South China Botanical Garden, Chinese Academy of Sciences), XTBG (Xishuangbanna Tropical Botanical Garden of Chinese Academy of Sciences), WBG (Wuhan Botanical Garden Chinese Academy of Sciences), DMNR (Diaoluo Mountain Nature Reserve, Hainan, China), NMNR (Nankun Mountain Nature Reserve, Huizhou, China), LMNR (Longgang Nature Reserve, Guangxi, China). ^b^ The voucher column lists the introduction number or specimen number of material used in this study. The numbers in parentheses indicate the number of plants.

**Table 2:** Taxon known to have both bicellular and tricellular pollen.

No.	Family	Taxon	% of Anthers with Both Types of Pollen	References
1	Annonaceae	*Uvaria grandiflora* Roxb	23.2% (±3%)	Present paper
2	Annonaceae	*Uvaria kurzii* (King) P. T. Li	55.0% (±5%)	Present paper
3	Annonaceae	*Uvaria calamistrata* Hance	33.3% (±5%)	Present paper
4	Annonaceae	*Annona cherimola* Mill	53.0% (±3%)	Present paper; [6]
5	Annonaceae	*Mitrephora wangii* Hu	33.3% (±5%)	Present paper
6	Annonaceae	*Mitrephora maingayi* Hook. f. et Thoms	33.3% (±5%)	[9]
7	Annonaceae	*Artabotrys hexapetalus* (L. f.) Bhandar	25.0% (±5%)	Present paper
8	Annonaceae	*Artabotrys hongkongensis* Hance	55.6% (±5%)	Present paper
9	Annonaceae	*Artabotrys pachypetalus* B.Xue & Junhao	50.0% (±3%)	Present paper
10	Annonaceae	*Goniothalamus calvicarpus* Craib	54.5% (±5%)	Present paper
11	Annonaceae	*Goniothalamus gardneri* Hook. f. et Thoms	52.5% (±3%)	Present paper
12	Annonaceae	*Goniothalamus chinensis* Merr. et Chun	53.5% (±5%)	Present paper
13	Annonaceae	*Fissistigma polyanthum* Hook. f. et Thoms	53.0% (±3%)	Present paper
14	Annonaceae	*Dasymaschalon rostratum* Merr. & Chun	50.0% (±2%)	Present paper
15	Annonaceae	*Meiogyne oligocarpa* B. Xue & Y. H. Tan	51.0% (±2%)	Present paper
16	Annonaceae	*Orophea laui* Leonardía & Kessler	53.0% (±5%)	Present paper
17	Annonaceae	*Polyalthia cheliensis* Hu	52.5% (±5%)	Present paper
18	Araceae	*Calla palustris*	——	[5]
19	Araceae	*Rhodospatha forgetii*	——	[5]
20	Araceae	*Anubias afzelii*	——	[5]
21	Araceae	*Dieffenbachia maculata*	——	[5]
22	Araceae	*Xanthosoma pilosum*	——	[5]
23	Araceae	*Chlorospatha castula*	——	[5]
24	Araceae	*Alocasia cuprea*	——	[5]
25	Asphodelaceae	*Hemerocallis* sp.	——	[28]
26	Asteraceae	*Conyza canadensis* (L.) C ronq	——	[29]
27	Berberidaceae	*Leontice incerta* Pall.	——	[13]
28	Berberidaceae	*Diphylleia sinensis* H. L. Li	——	[30]
29	Euphorbiaceae	*Beyeria leschenaultii*	——	[4]
30	Gentianaceae	*Swertia bimaculata*	——	[31]
31	Gentianaceae	*Tripterospermum chinense (Migo) Harry Sm.*	——	[32]
32	Lauraceae	*Laurelia novae-zelandiae* A. Cunn	——	[33]
33	Lauraceae	*Beilschmiedia tara*	——	[34]
34	Lauraceae	*Beilschmiedia taw*	——	[34]
35	Magnoliaceae	*Michelia figo* (Lour.) Spreng.	——	[15]
36	Plumbaginaceae	*Limonium* sp.	——	[35]
37	Poaceae	*Bambusa textilis*	——	[14]
38	Poaceae	*Shibataea chinensis*	——	[36]
39	Poaceae	*Arundinaria simonii* f. albostriatus	——	[36]
40	Poaceae	*Pseudosasa viridula*	——	[12]
41	Poaceae	*Menstruocalamus sichuanensis*	——	[36]
42	Poaceae	*Bambusa multiplex*	——	[8]
43	Poaceae	*Sasaella kogasensis* ‘Aureostriatus’	——	[11]
44	Poaceae	*Phyllostachys edulis* (Carrière) J. Houzeau	——	[16]
45	Ranunculaceae	*Adonis amurensis* Regel et Radde.	——	[10]
46	Ranunculaceae	*Coptis deltoidea* C. Y. Cheng et Hsiao	——	[37]
47	Saxifragaceae	*Saxifraga pseudohirculus*	——	[7]
48	Saxifragaceae	*Saxifraga caveana*	——	[7]
49	Solanaceae	*Solanum phureja*	——	[38]
50	Solanaceae	*Solanum japonense* Nakai	——	[39]
51	Solanaceae	*Solanum septemlobum* Bunge	——	[39]
52	Violaceae	*Viola tricolor* L.	——	[40]

## Data Availability

The original contributions presented in this study are included in the article. Further inquiries can be directed at the corresponding author.
